# Editorial: Financial intermediation versus disintermediation: opportunities and challenges in the FinTech era, volume II

**DOI:** 10.3389/frai.2024.1326358

**Published:** 2024-01-19

**Authors:** Anna Omarini

**Affiliations:** Department of Finance, Bocconi University, Milan, Italy

**Keywords:** digital transformation, digital finance, FinTech, contextual banking, embedded banking, ecosystem, modularity, personalization

Digital transformation has become a key priority for many banks seeking to remain competitive and meet the evolving demands of customers. However, new technologies that fundamentally change the business models and operations of banks pose new threats and opportunities in the digital vortex. These challenges can be described as the inevitable transition of industries toward a digital center in which business models, offerings, and value chains are maximally digitized, creating disruptions and blurring the lines between industries.

Digitalization is changing the rules of many industries, resulting in the emergence of complex and dynamic ecosystems for growth and innovation.

The main forces shaping these changes have led the financial service industry to reconsider the roles of banking and finance as enablers as opposed to merely providers of products and services.

The aforementioned changes are forcing the financial service industry to continuously undergo and manifest new trends. Banks are the entities most affected by these changes, as their future lies in the needs of their customers. These needs are themselves subject to important changes stemming from the new value propositions of Big Tech and financial technology (FinTech), which encourage switching and comparisons with improved results and product offerings for consumers.

The concept of FinTech first emerged with the identification of the main technological drivers of change–big data analytics, artificial intelligence, and blockchain technology–and their financial applications in the context of the primary functions of banks. These applications include maturity transformation (through competition in lending), allocation (through robot-advisor and crowd-investing platforms), information processing (through big data, machine learning, and AI), liquidity provision, and risk pooling.

In contrast, Big Tech companies provide banking and other financial services alongside the feature of being intrinsically linked to the rise of big data, data analytics, and related opportunities.

All of these have brought a wave of competition and broken pipeline value chains to the traditional banking industry, unbundling them into different modules of products and services that may be combined among themselves.

We are currently in the early stages of transforming the banking sector and implementing new technologies. Consequently, both regulators and supervisors face the challenges introduced by digital transformation, necessitating an appropriate balance between promoting new digital value propositions and protecting against the risks inherent to the digitalization of financial services. The aforementioned scenarios are associated with both conventional and novel risks. The latter originate from the increasing use of big data, robot-advisor platforms, AI, machine learning, and other tools deployed to enhance customer personalization and user experience.

The linkage of digital finance to real economic technologies must be sustainable while minimizing the potential negative impacts on consumers and investors. This goal can be achieved through the development of appropriate risk management methods whose compliance burdens can be limited by the technology itself (see Giudici).

It is also worth noting that financial services rely on credence qualities and are highly trust-driven. This tendency is demonstrated by the effect of robot-facilitated services on the trust provided to customers (see Giudici).

Consequently, several innovations have gained traction in the market from a technological perspective. The cloud – particularly in the cases of Platform as a Service (PaaS) and Software as a Service (SaaS) – is lowering the barrier to sophisticated financial applications by allowing people and talent to focus on business-value-added tasks as opposed to building, supporting, and managing infrastructure. Advanced AI and machine-learning techniques also allow data scientists and researchers to reveal non-obvious patterns in complex high-dimensional data.

Regulatory technology (RegTech), another interesting application of FinTech, is expected to not only provide significant efficiency gains for compliance and reporting functions, but also considerably change market structure and supervision (see Gasparri).

New banking will emerge with richer ecosystems, wherein the deep deintegration of financial solutions will emerge through embedded and contextual banking, increasing customer loyalty. Both are the new trends in banking. Both embedded and contextual banking represent novel trends rooted in the increasing prevalence of modularity, which lies in the ability of companies to move toward product componentization. The important benefits of modularity are customization and personalization, whereas its novelty comes from the digitalization process, which has resulted in an array of different use cases boosted by application programming interface technology.

The new current outlook reveals nascent ecosystems composed of independent actors, where the traditional supply-centered oligopoly is coupled with FinTech, TechFin, and retailers. Within this system lies the disruptive aspect of PSD2 in Europe, as well as similar trends in other markets. This is a key milestone in the unbundling and modularization of banking and non-banking services, challenging the financial service landscape.

Banking is shifting from a pipelined vertical paradigm to an open model. Consequently, open innovation, modularity, and ecosystem-based banking may come to embody the mainstream paradigm. However, many new opportunities exist for banks to regain their role in the market through a reintermediation process.

The primary unique characteristic of the present situation is the relationship between technology and banking, which has stronger interdependencies from a double perspective: technological and, more importantly, strategic interdependence.

## Author contributions

AO: Conceptualization, Writing – review & editing.

